# Novel Mobilizable Genomic Island GEI-D18A Mediates Conjugational Transfer of Antibiotic Resistance Genes in the Multidrug-Resistant Strain *Rheinheimera* sp. D18

**DOI:** 10.3389/fmicb.2020.00627

**Published:** 2020-04-07

**Authors:** Jiafang Fu, Chuanqing Zhong, Peipei Zhang, Gongli Zong, Meng Liu, Guangxiang Cao

**Affiliations:** ^1^Department of Epidemiology, The First Affiliated Hospital of Shandong First Medical University, Jinan, China; ^2^Shandong Medicinal Biotechnology Center, Shandong First Medical University, Shandong Academy of Medical Sciences, Jinan, China; ^3^School of Municipal and Environmental Engineering, Shandong Jianzhu University, Jinan, China; ^4^Key Laboratory for Biotech-Drugs of National Health Commission, Jinan, China

**Keywords:** *Rheinheimera*, mariculture, multidrug resistance, resistance genomic island, conjugation

## Abstract

Aquatic environments act as reservoirs of antimicrobial-resistant bacteria and antimicrobial resistance (AMR) genes, and the dissemination of antibiotic resistance from these environments is of increasing concern. In this study, a multidrug-resistant bacterial strain, identified as *Rheinheimera* sp. D18, was isolated from the sea water of an industrial maricultural system in the Yellow Sea, China. Whole-genome sequencing of D18 revealed the presence of a novel 25.8 kb antibiotic resistance island, designated GEI-D18A, which carries several antibiotic resistance genes (ARGs), including *aadA1*, *aacA3*, *tetR*, *tet*(B), *catA*, *dfrA37*, and three *sul1* genes. Besides, integrase, transposase, resolvase, and recombinase encoding genes were also identified in GEI-D18A. The transferability of GEI-D18A was confirmed by mating experiments between *Rheinheimera* sp. D18 and *Escherichia coli* 25DN, and efflux pump inhibitor assays also suggested that *tet*(B) in GEI-D18A was responsible for tetracycline resistance in both D18 and the transconjugant. This study represents the first characterization of a mobilizable antibiotic resistance island in a species of *Rheinheimera* and provides evidence that *Rheinheimera* spp. could be important reservoirs and vehicles for ARGs in the Yellow Sea area.

## Introduction

In aquaculture systems, antibiotics are commonly applied with feed or directly to the water, and may later be released into the environment (Fortt et al., 2007; Buschmann et al., 2012; Muziasari et al., 2017). Therefore, the aquaculture environment could become an important source of antimicrobial resistance (AMR) bacteria and AMR genes that could pose risks to human health (Garcia-Aljaro et al., 2014; Xu et al., 2017). The Yellow Sea area is an important aquaculture site along the coastline of China, and antibiotic resistance genes (ARGs) have become an emerging form of environmental pollution in this marine area (Na et al., 2014). The top three ARGs of highest abundances in the Yellow Sea area are sulfonamide resistance genes (*sul*), tetracycline resistance genes (*tet*) and quinolone resistance genes (*qnr*), and *tetG*/*tetX* and *sul1* genes have strong correlations with *Aeromicrobium, Lysobacter*, *Blastomonas* (Lu et al., 2019). However, there have been no studies investigating the ability of ARGs to transfer and generate new antibiotic-resistant strains in the Yellow Sea area, but such studies are needed for understanding potential new sources of antibiotic resistance.

Tetracycline has been used widely to treat bacterial infections in aquaculture environment (Kim et al., 2004; Gao et al., 2012), and tetracycline resistance genes (*tet*) are one of the highest abundances in Yellow Sea area (Lu et al., 2019). Therefore, in this report, in order to investigate the transfer of ARGs such as tetracycline resistance genes in the Yellow Sea area, a multidrug-resistant strain, identified as *Rheinheimera* sp. D18, was isolated using tetracycline as screening pressure from the seawater of an industrial mariculture system. The genus *Rheinheimera*, which was first described by Brettar et al. (2002), is Gram-stain-negative, flagellated, rod-shaped to coccoid, aerobic bacterial cell that is commonly isolated from surface and deep seawater (Brettar et al., 2002; Yoon et al., 2007). Many strains of *Rheinheimera* are resistant to tetracycline, rifampicin, chloramphenicol, ciprofloxacin, ampicillin, vancomycin, gentamicin, penicillin, streptomycin, polymyxin, novobiocin, or other antibiotics (Liu et al., 2012; Mengoni et al., 2014; Suarez et al., 2014; Kumar et al., 2015). Genome sequencing and bioinformatic analysis have uncovered a series of ARGs in the genome of *Rheinheimera* sp. EpRS3, such as an *acrD* homolog encoding an aminoglycoside efflux pump; the *acrAB, mexGHI*, and *mdtABC* genes, which encode subunits of the AcrAB/TolC, MexGHI-OpmD, and MdtABC-TolC efflux pumps, respectively; and other genes encoding complete RND-type efflux pumps, suggesting that *Rheinheimera* sp. EpRS3 is a multidrug-resistant strain (Moore et al., 1999; Rosenberg et al., 2000; Presta et al., 2017), while the transferability of ARGs in *Rheinheimera* remains unclear.

In this report, a mobilizable genomic island (named GEI-D18A), which contains a TetR-family transcriptional regulator, the tetracycline efflux MFS transporter Tet(B), as well as several other ARGs, was identified in the genome of *Rheinheimera* sp. D18. This study represents the first characterization of a mobilizable genomic island that confers multidrug resistance in the genus *Rheinheimera*, providing evidence that *Rheinheimera* spp. could be important reservoirs and vehicles for drug resistance genes. Additionally, our study provides new insights into ARG dissemination in the Yellow Sea area, an area of substantial aquaculture importance.

## Materials and Methods

### Bacterial Strains, Isolation, and Growth Conditions

The seawater sample from an industrial mariculture system in the Yellow Sea, China, was serially diluted with sterilized water, plated onto LB (0.5% yeast extract, 1% tryptone, 1% sodium chloride, 2% agar) solid medium supplemented with 16 mg/L tetracycline, and then incubated at 28°C for 24 h to obtain single colonies. Then, one of the single colonies was picked and streaked onto LB solid medium containing tetracycline in order to obtain a pure culture. After further purification, a single colony was selected and grown as a pure culture, which was named strain D18. *Escherichia coli* strains were grown in LB medium at 37°C.

### Identification and Phylogenetic Affiliation Analysis of Strain D18

To identify the species of strain D18, a partial fragment of 16S rDNA was amplified with the primer pair 27F (5′-AGAGTTTGATCCTGGCTCAG-3′) and 1492R (5′-GGTTACCTTGTTACGACTT-3′), and then DNA sequencing was performed for preliminary identification.

**FIGURE 1:**
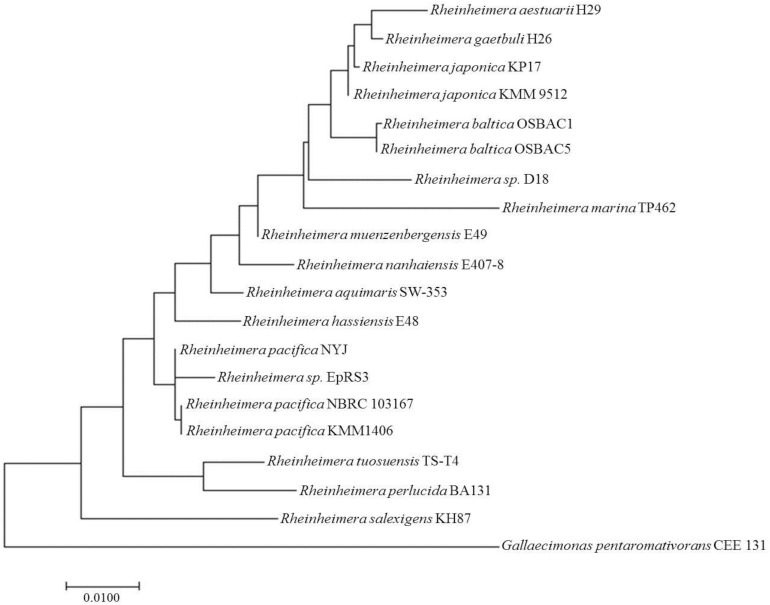
Molecular phylogenetic analysis of *Rheinheimera* sp. D18 based on 16S rRNA gene sequences. Eighteen 16S rRNA gene sequences belonging to *Rheinheimera* species were selected, the 16S rRNA gene sequence from *Gallaecimonas pentaromativorans* CEE 131 was used as an outgroup, and the phylogenetic tree was inferred using the maximum likelihood method based on the Tamura-Nei model.

Eighteen 16S rRNA gene sequences belonging to *Rheinheimera* species (representing all the species sequences available for this genus) were selected from the NCBI FTP site. Moreover, the 16S rRNA gene sequence from *Gallaecimonas pentaromativorans* CEE 131 was included as an outgroup in phylogenetic analysis. The phylogenetic tree was inferred by using the maximum likelihood method based on the Tamura-Nei model (Tamura and Nei, 1993). The tree with the highest log likelihood (−4017.73) is shown (Figure 1). Initial trees for the heuristic search were obtained automatically by applying neighbor-joining and BioNJ algorithms to a matrix of pairwise distances estimated using the maximum composite likelihood (MCL) approach, and then selecting the topology with superior log likelihood value. The phylogenetic tree is drawn to scale, with branch lengths measured in the number of substitutions per site. The analysis involved 24 nucleotide sequences, and the included codon positions were 1st + 2nd + 3rd + Non-coding. All positions containing gaps and missing data were eliminated. There were a total of 1,301 positions in the final dataset. Evolutionary analyses were conducted in MEGA7 (Kumar et al., 2016).

### Whole-Genome Sequencing, Genomic Annotation, and Analysis

Strain D18 was cultured in LB medium at 28°C, 200 rpm, and then the genomic DNA was extracted using the Genomic DNA Purification Kit (Promega, United States). The quality of the genomic DNA sample was analyzed using a NanoDrop spectrophotometer (Thermo Scientific, United States). The genome of D18 was sequenced and assembled with a PacBio RS II platform and an Illumina NextSeq platform at BGI Co., Ltd. (Wuhan, China). Genome annotation was performed using the Prokaryotic Genome Annotation Pipeline (PGAP) on NCBI^1^. Additional genome annotation was performed using the Pathosystems Resource Integration Center (PATRIC) server (Wattam et al., 2017) and using the RASTtk server (Overbeek et al., 2014; Brettin et al., 2015). Genomic islands (GIs) were predicted by IslandViewer 4 (Bertelli et al., 2017). To determine if GEI-D18A belongs to ICE (Integrative and Conjugative Element), the whole nucleotide sequence of GEI-D18A was detected by ICEfinder (Liu et al., 2019). IS transposase were detected by IS-Finder (Siguier et al., 2006). The integron were predicted by INTEGRALL website (Moura et al., 2009). Sequence alignment was performed by BLAST server^2^. The antiSMASH software (Medema et al., 2011) was used to analyze the secondary metabolite biosynthetic gene clusters of the *Rheinheimera* sp. D18 genome.

### Mating Assays

To determine if the antibiotic resistant genes could be transferred from *Rheinheimera* sp. D18 to other bacteria by horizontal gene transfer (HGT), mating experiments between D18 and the sodium azide-resistant *E. coli* 25DN strain (derived from *E. coli* ATCC 25922) were performed as previously described (Li et al., 2019). The D18 strain and *E. coli* 25DN were mixed, with or without DNase I (50 mg/L), and cultured on LB solid medium containing tetracycline, sodium azide and X-Gluc (5-bromo-4-chloro-3-indolyl-beta-D-glucuronic acid) to screen the transconjugants. D18 (donor) and *E. coli* 25DN (recipient) are inhibited by sodium azide and tetracycline, respectively, and only the transconjugants of *E. coli* can survive on the selective medium and degrade X-Gluc to blue compounds. The episomal form of GEI-D18A in *Rheinheimera* sp. D18, and GEI-D18A in the transconjugants were verified by PCR (primers are listed in Supplementary Table S1) and DNA sequencing.

### Determination of the Minimum Inhibitory Concentrations (MICs) With and Without Efflux Pump Inhibitor

To determine the MICs of *Rheinheimera* sp. D18 and the above transconjugant strain for different antibiotics, including ampicillin, cefixime, ciprofloxacin, tetracycline, amikacin, florfenicol, sulfamethoxazole, polymyxin E, and meropenem, the broth microdilution method was used, as described by the Clinical Laboratory Standard Institute (CLSI) guidelines (CLSI, 2017).

The MICs for tetracycline in the presence of efflux pump inhibitors were calculated by broth microdilution procedure. Carbonyl cyanide m-chlorophenylhydrazone (CCCP) and verapamil were used as efflux pump inhibitors at final concentration of 0.1 and 80 mg/L, respectively (Jain et al., 2016; Ni et al., 2016). *E. coli* 25DN strain was used in each test for internal quality control.

## Results

### Bacterial Strain Identification and General Genomic Properties of *Rheinheimera* sp. D18

Strain D18 was isolated from the seawater of an industrial mariculture system in the Yellow Sea, China. The phylogenetic tree based on the 16S rRNA gene sequence shows that strain D18 is within a subcluster of the genus *Rheinheimera* (Figure 1). D18 positioned within the genus *Rheinheimera* (class *Gammaproteobacteria*) as a separate subline adjacent to *Rheinheimera baltica*. *Rheinheimera* sp. D18 exhibited 96.84% 16S rDNA nucleotide sequence identity with the *R. baltica* type strain OSBAC5.

The whole genome of *Rheinheimera* sp. D18 was sequenced, and the general features of the complete genome sequence were analyzed (Supplementary Figure S1). D18 harbors a circular chromosome of 3,308,353 bp, with an average GC content of 44.39%. The annotation indicated that the genome contained genes for 2,961 proteins; 15 rRNA genes; 69 tRNA genes; four other RNA genes and 58 pseudogenes. In addition, three gene clusters associated with the biosynthesis of hserlactone and arylpolyene were identified in the genome of D18 using the antiSMASH suite (Supplementary Table S2), suggesting that D18 strain has potential antimicrobial activity.

### Susceptibility Testing and Antimicrobial Resistance Genes in Strain D18

Susceptibility testing revealed that *Rheinheimera* sp. D18 is resistant to at least nine different antibiotics, including ampicillin, cefixime, ciprofloxacin, tetracycline, amikacin, florfenicol, sulfamethoxazole, polymyxin E, and meropenem. The MICs of the above antibiotics for strain D18 are shown in Table 1. The results showed that strain D18 had striking resistance to tetracycline (MIC ≥ 160 mg/L), amikacin (MIC ≥ 96 mg/L), florfenicol (MIC ≥ 128 mg/L), and sulfamethoxazole (MIC ≥ 72 mg/L).

**TABLE 1:** MICs of antibiotics for strain *Rheinheimera* sp. D18 and *E. coli* transconjugant 25D18-T2.

Strain	Ampicillin*	Cefixime	Ciprofloxacin	Tetracycline	Amikacin	Florfenicol	sulfamethoxazole	Polymyxin E	Meropenem
D18	36	16	34	160	96	128	72	18	26
25DN	8	<2	<2	4	<2	<2	4	<2	<2
25D18-T2	8	<2	<2	72	36	64	68	<2	<2

*Concentration units for the antibiotics tested: mg/L.

Dozens of ARGs were identified in the D18 genome (Supplementary Data Sheet S1), including genes associated with resistance to tetracycline [*tet(B)*]; sulfonamides (*sul1*, *sul2*); chloramphenicol/florfenicol (*floR*, *catA2*); aminoglycoside (*aphA1*, *aphA6*, *aacA3*, *aadA1*); macrolides (*macA*); penicillin (*lpoA*); fosmidomycin (*hdN1*), and antibiotic efflux pump encoding genes. The identification of numerous ARGs partially illuminated the basis for the extreme multidrug resistance trait of strain D18. It is worth noting that strain D18 had extremely high resistance to tetracycline (MIC ≥ 160 mg/L). Notably, only one known tetracycline resistance gene, *tet(B)*, which encodes the tetracycline resistance major facilitator superfamily (MFS) efflux pump (E0Z06_RS15040), and the *tetR* gene, encoding the TetR-family transcriptional regulator, were predicted in the genome of D18. We hypothesize that TetB efflux pump is major factor in the high level of tetracycline resistance of *Rheinheimera* sp. D18.

### Identification and Description of a New Resistance Genomic Island GEI-D18A

**FIGURE 2:**
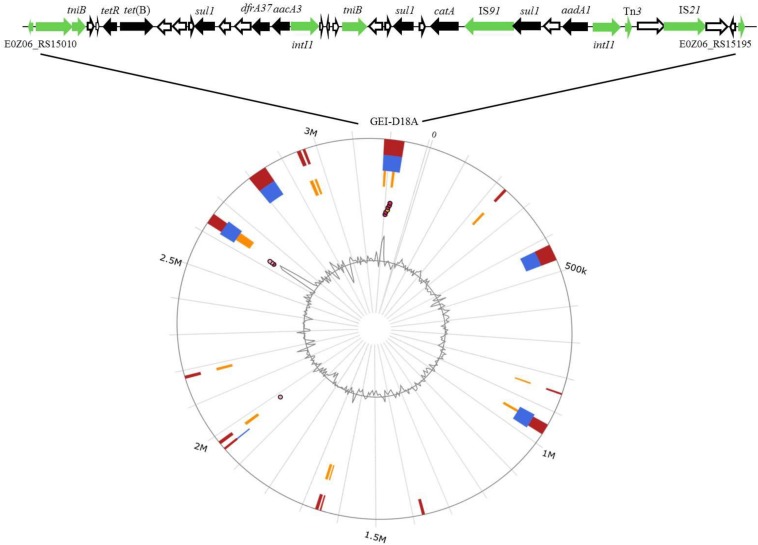
Identification of GEI-D18A in the genome of D18. Top line, gene arrangement in GEI-D18A. Genes are denoted by arrows. Antibiotic resistance genes, black arrows; conjugational transfer-related genes (transposase, resolvase, integrase, and recombinase encoding genes), green arrows; and genes of unknown function, white arrows. Bottom image, GIs predicted by IslandViewer 4. Putative GIs predicted by the SIGI-HMM method (yellow squares) or IslandPath-DIMOB method (blue squares). The integrated results are indicated by red squares.

**FIGURE 3:**
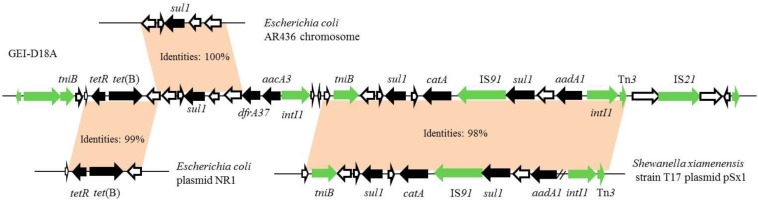
Map showing similarities between GEI-D18A antibiotic resistance gene regions and other strains. GEI-D18A sequence was compared with other strains using Standard Nucleotide BLAST online website (https://blast.ncbi.nlm.nih.gov/Blast.cgi) and the top three matches (*Escherichia coli* AR436, *Escherichia coli* plasmid NR1 and *Shewanella xiamenensis* T17 plasmid pSx1) were presented. Antibiotic resistance genes, black arrows; transposase, resolvase, integrase, and recombinase encoding genes, green arrows; genes of unknown function, white arrows.

The GIs in D18 were predicted by IslandViewer 4 software combined with genome annotation (Figure 2). Two overlapping regions, the first ranging from nucleotide positions 3,183,596–3,238,484 and the second from positions 3,214,442 to 3,222,752, were respectively annotated as potential antibiotic GIs by IslandPath-DIMOB and SIGI-HMM. Further alignment analysis indicated that the antibiotic GI most likely extends from position 3,200,681 to 3,226,474, and this 25,794 bp region was named GEI-D18A. The GC content of GEI-D18A is 52.26%, which differs from that of the overall genome (44.39%), suggesting that GEI-D18A was obtained from other bacteria. In addition, BLAST Alignment showed that the GEI-D18A is only present in *Rheinheimera* sp. D18 genome, but not other sequenced strains of the genus *Rheinheimera*. Sequence examination further suggested that the integration of GEI-D18A occurred within the intergenic sequence between E0Z06_RS15010 and E0Z06_RS15195 and that it resulted in the 12-bp direct repeat (DR) GATCTGCANNAA flanking GEI-D18A. Thirty-six ORFs were identified in GEI-D18A by genomic annotation, including night ARGs associated with tetracycline [*tetR* and *tet*(B)], sulfonamide (three *sul1* genes), aminoglycoside (*aadA1* and *aacA3*), trimethoprim (*dfrA37*) and chloramphenicol/florfenicol (*catA*) resistance. In addition, the three transposase encoding genes E0Z06_RS15145, E0Z06_RS15170 and E0Z06_RS15180; two integrase encoding genes, E0Z06_RS15085 and E0Z06_RS15165; one DDE-type integrase/transposase/recombinase encoding gene E0Z06_RS15015; one recombinase encoding gene E0Z06_RS15195; one resolvase encoding gene E0Z06_RS15010; and two ATP-binding protein TniB (involved in transposition) encoding genes, E0Z06_RS15020 and E0Z06_RS15105, were identified in GEI-D18A, suggesting that GEI-D18A can potentially transfer into other bacteria via horizontal gene transfer. Further sequence comparisons revealed that partial genes in GEI-D18A were highly similar with those of *Escherichia coli* AR436, *Escherichia coli* plasmid NR1 and *Shewanella xiamenensis* T17 plasmid pSx1 (Figure 3), indicating that *Rheinheimera* sp. D18 acquire GEI-D18A from other bacterial species through HGT.

### GEI-D18A Can Be Conjugational Transferred From D18 to *E. coli*

**FIGURE 4:**
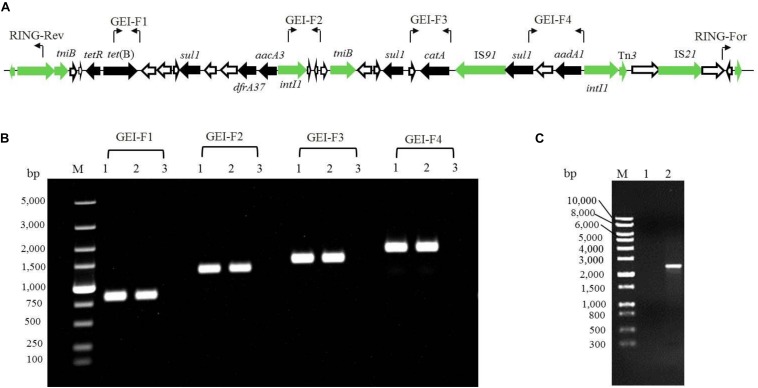
Verification of the presence of GEI-D18A by PCR. **(A)** PCR primer positions in GEI-D18A are indicated by bent arrows. Antibiotic resistance genes in GEI-D18A, black arrows; conjugational transfer-related genes, green arrows; and genes of unknown function, white arrows. **(B)** GEI-D18A regions were amplified by PCR using the following templates: lane 1, total DNA of conjugant 25D18-T2; lane 2, total DNA of strain D18; lane 3, total DNA of strain 25DN. M, molecular size markers. **(C)** Gel picture of the PCR product generated by the RING-For and RING-Rev primers. M, DNA marker; lane 1, total DNA of strain *E. coli* 25DN used as template; lane 2, total DNA of strain *Rheinheimery* sp. D18 used as template.

To determine the transferability of GEI-D18A from the D18 genome, mating experiments between D18 (donor) and *E. coli* 25DN (recipient) were carried out. The transconjugants were selected on tetracycline plates, and results revealed that the transconjugation frequency between D18 and 25DN was 6.4 × 10^–7^ CFU/donor. One of the transconjugants was isolated and named 25D18-T2. MIC testing revealed that the MIC for tetracycline in the transconjugant 25D18-T2 was 72 mg/L, much weaker than that of the donor strain D18 (160 mg/L) but much higher than the MIC for the recipient strain 25DN (Table 1). In addition, 25D18-T2 also acquired resistance to amikacin (MIC ≥ 36 mg/L), florfenicol (MIC ≥ 64 mg/L), and sulfamethoxazole (MIC ≥ 68 mg/L). The obvious increase in multiple antibiotic resistance of the transconjugant 25D18-T2 compared to 25DN suggested that the ARGs in D18 had transferred to *E. coli* via conjugation. PCR and DNA sequencing analysis confirmed that GEI-D18A had been transferred to 25D18-T2, and GEI-D18A excised from the chromosome to form a circular extrachromosomal molecule in *Rheinheimera* sp. D18 (Figure 4), indicating that GEI-D18A is responsible for the multiple antibiotic resistance of 25D18-T2. In addition, the same transconjugation frequency was observed when mating experiments were performed in the presence of DNase I (50 mg/L), which suggests that GEI-D18A was transferred from D18 to *E. coli* 25D18-T2 by conjugation rather than transformation.

### Effects of Efflux Pump Inhibitors on Tetracycline Resistance

Resistance to tetracycline can be associated with efflux pumps, ribosomal protection systems and/or other mechanisms (Rolain, 2013). The whole genome sequence of *Rheinheimera* sp. D18 revealed the presence of only one tetracycline efflux pump encoding gene, *tet(B)*. The efflux pump TetB belongs to the major facilitator superfamily (Wang et al., 2017). TetB is energized by the proton motive force, therefore, CCCP and verapamil, inhibitors of the proton motive force, are widely used to study the bacterial efflux pump (Andersen et al., 2006; Ikonomidis et al., 2008; Anoushiravani et al., 2009). In this report, in order to evaluate efflux-mediated tetracycline resistance, the tetracycline-resistant strains *Rheinheimera* sp. D18 and *E. coli* transconjugant 25D18-T2 were subjected to MIC determination in the presence of the efflux pump inhibitors CCCP and verapamil. The presence of CCCP, an inhibitor of the MFS proteins, in broth at a subinhibitory concentration (0.1 mg/L), reduced the MIC for tetracycline from 160 to 50 mg/L for strain D18. CCCP also reduced the MIC for tetracycline from 72 to 24 mg/L for strain 25D18-T2 (Table 2). As shown in Table 2, 80 mg/L verapamil had a similar effect, resulting in decreased MICs of tetracycline. The effects of these efflux pump inhibitors on tetracycline resistance further suggested that the 25D18-T2 strain acquired tetracycline resistance by obtaining the *tet(B)* gene in GEI-D18A.

**TABLE 2:** Effects of efflux pump inhibitors on MICs of tetracycline.

Strain	Efflux pump inhibitor	MIC (mg/L)
D18	–*	160
	CCCP	50
	Verapamil	32
25D18-T2	–	72
	CCCP	24
	Verapamil	16

*No efflux pump inhibitor was added.

## Discussion

In addition to plasmid-mediated transformation and conjugation, and phage-mediated transduction, GEIs are an important means of bacterial HGT (Dobrindt et al., 2004; Juhas et al., 2009). GEIs are typically characterized as discrete DNA segments that reside on the chromosome close to a tRNA gene and carry an integrase encoding gene and a short duplication of the insertion site at the other end; some GEIs are mobile whereas others are not or are no longer mobile (Hacker and Carniel, 2001). GEIs can be further divided into many subclasses, including pathogenicity islands and antibiotic resistance islands. Many GEIs are or can be integrated into the chromosome of the host, excised, and transferred to other recipients by transformation, conjugation or transduction (Dobrindt et al., 2004). Therefore, GEIs can have a significant impact on the dissemination of ARGs and contribute to pathogenicity and primary and secondary metabolism (Dobrindt et al., 2004).

Antibiotic resistance genes can be transferred between bacterial strains by plasmids or phage. However, *Rheinheimera* sp. D18 harbors only a circular chromosome, and no plasmids or phages were identified. Nevertheless, mating experiments revealed that tetracycline, florfenicol, amikacin, and sulfonamide resistance genes in *Rheinheimera* sp. D18 could be transferred to *E. coli* 25DN via HGT. Further analysis of the D18 genome sequence identified a novel antibiotic resistance island, which we named GEI-D18A. In addition to ARGs, GEI-D18A also contains genes encoding transposases, recombinases, resolvase, and transposition proteins (Supplementary Table S3), whereas ICEfinder analysis revealed that the mobilizable genomic island GEI-D18A is not an ICE. Notably, there are four relaxase encoding genes and 20 type IV secretion system encoding genes (Supplementary Data Sheet S2) in the D18 genome. Adapted conjugation, mediated by type IV secretion systems, is also widespread in nature (Christie and Vogel, 2000; Christie, 2001; Juhas et al., 2007). Since type IV secretion systems can mediate HGT, we propose that GEI-D18A was transferred from *Rheinheimera* sp. D18 to *E. coli* 25DN via a type IV secretion system: firstly, the GEI-D18A was excised from the *Rheinheimera* sp. D18 chromosome to form a circular extrachromosomal molecule (Figure 4C). Then, the episomal form of GEI-D18A was transferred to the recipient *E. coli* 25DN strain via type IV secretion system with the help of series of conjugational transfer-related elements. Finally, the GEI-D18A may be integrated into the *E. coli* 25DN at short sequences that are nearly identical to the 12-bp direct repeat sequence (GATCTGCANNAA) flanking GEI-D18A as *E. coli* 25DN harbors dozens of this above consensus sequence in genome. In addition, GEI-D18A may also be transferred from *Rheinheimera* sp. D18 to *E. coli* 25DN through other mechanisms that remain to be investigated.

An abundance of multidrug-resistant strains have been detected in the Yellow Sea (Lu et al., 2019). The *sul1* gene is usually located in a class 1 integron, which is considered to be the main mobile genetic element to transfer *sul1* among strains in the Yellow Sea (Na et al., 2014). In the multidrug-resistant strain *Rheinheimera* sp. D18, isolated in this report, three copies of *sul1* and other ARGs were located on GEI-D18A. The complete genome of the *Rheinheimera* sp. D18 has been analyzed using INTEGRALL, analysis revealed that GEI-D18A contains two class 1 integrons In1679 and In2 (Supplementary Data Sheet S3), and both the two integrons include *sul1* gene. In addition, In1679 contains three gene cassettes: *aacA3, dfrA37, orf*, In2 contains one gene cassette: *aadA1*. Moreover, In1679 is firstly detected in *Rheinheimera*. Furthermore, mating assays revealed that GEI-D18A can be conjugational transferred between bacterial strains, yielding a new multiple-antibiotic-resistant strain. However, the transferability of GEI-D18A from the *E. coli* transconjugant has not been determined, and the propagation ability of ARGs in this island needs to be further investigated.

In conclusion, the whole-genome sequencing of the multidrug-resistant *Rheinheimera* sp. D18 revealed the presence of a novel 25.794 kb antibiotic resistance island, named GEI-D18A, which contains nine ARGs, three transposase encoding genes, one resolvase encoding gene, two integrase encoding genes, one DDE-type integrase/transposase/recombinase encoding gene, one recombinase encoding gene and two ATP-binding protein (involved in transposition) encoding genes. Mating assays demonstrated that GEI-D18A could be transferred from *Rheinheimera* sp. D18 to *E. coli* 25DN, and we propose that this transfer may have occurred via a type IV secretion system. This study identifies the first mobilizable antibiotic resistance island in a *Rheinheimera* species. However, the mechanisms by which GEI-D18A can be transferred to other bacterial strains remain to be investigated.

## Data Availability Statement

The datasets generated for this study can be found in the whole genome sequence of *Rheinheimera* sp. D18 was deposited in GenBank under accession No. CP037745. The 16S rDNA sequence of strain D18 was submitted to GenBank and was assigned accession No. MN726625.

## Author Contributions

GC designed the work and revised the manuscript. JF executed the experiments, manuscript preparation and submission. PZ and ML carried out the interpretation of data. CZ contributed to the experimental design. GZ helped in data analysis. All authors read and approved the final manuscript.

## Conflict of Interest

The authors declare that the research was conducted in the absence of any commercial or financial relationships that could be construed as a potential conflict of interest.

## Footnotes

1 ncbi.nlm.nih.gov/genome/annotation_prok/

2 https://blast.ncbi.nlm.nih.gov/Blast.cgi

## Supplementary Material

FIGURE S1 Comprehensive genomic analysis of *Rheinheimera* sp. D18.

TABLE S1 Primers used in this study.

TABLE S2 Gene clusters involved in secondary metabolite biosynthesis in the *Rheinheimera* sp. D18 genome.

TABLE S3 Genes annotated in GEI-D18A.

DATA SHEET S1 Antibiotic resistance genes in *Rheinheimera* sp. D18.

DATA SHEET S2 Type IV secretion system encoding genes in *Rheinheimera* sp. D18.

DATA SHEET S3 The detailed information about the structure of two integrons In2 and In1679.
