# Supplementary material for: Novel Mobilizable Genomic Island GEI-D18A Mediates Conjugational Transfer of Antibiotic Resistance Genes in the Multidrug-Resistant Strain Rheinheimera sp. D18
Source: Front Microbiol. 2020 Apr 7;11:627. doi: 10.3389/fmicb.2020.00627 (PMC7155750; doi:10.3389/fmicb.2020.00627)
Supplement: FIGURE S1 — Comprehensive genomic analysis of Rheinheimera sp. D18. [file Data_Sheet_4.docx]

**Supplementary Material**

**Novel Mobilizable Genomic Island GEI-D18A Mediates Conjugational Transfer of Antibiotic Resistance Genes in the Multidrug-resistant Strain** ***Rheinheimera* sp. D18**

Jiafang Fu^1, 2^, Chuanqing Zhong^3^, Peipei Zhang^1,2^, Gongli Zong^1,4^, Meng Liu^3^, Guangxiang Cao^1,2^*

^1^ Department of Epidemiology, the First Affiliated Hospital of Shandong First Medical University, Jinan 250062, China

^2^ Shandong Medicinal Biotechnology Center, Shandong First Medical University & Shandong Academy of Medical Sciences, Jinan 250062, China

^3^ School of Municipal and Environmental Engineering, Shandong Jianzhu University, Jinan 250101, China

^4^ Key Laboratory for Biotech-Drugs of National Health Commission, Jinan 250062, China

**TABLE S1** Primers used in this study.

| **Oligonucleotide** | **DNA Sequence (5’→3)** |
| --- | --- |
| 27F | AGAGTTTGATCCTGGCTCAG |
| 1492R | GGTTACCTTGTTACGACTT |
| GEI-F1-For(3204812) | ATTCCCGCAACGGTGTGGGTGCT |
| GEI-F1-Rev(3205653) | TAAGGTAAACGCCATTGTCAGCA |
| GEI-F2-For(3210015) | CACCCGGCAACCTTGGGCAGCAG |
| GEI-F2-Rev(3211194) | ACTTATTGGATATTTGGAATAGG |
| GEI-F3-For(3214601 | CGGTAAGGCCATTGTGACACTG |
| GEI-F3-Rev(3216284) | GTGTGCTGCATCCGGAAGTGTC |
| GEI-F4-For(3222802) | TGAGTGCATAACCACCAGCCTG |
| GEI-F4-Rev(3224783) | GCCGGGTGACGCACACCGTGGA |
| RING-For(3225198) | CGGTCAGAAGCGACCTGACTAC |
| RING-Rev(3202127) | GGCGTAGTAGTGGATGTGGTCG |

**TABLE S2** Gene clusters involved in secondary metabolite biosynthesis in the *Rheinheimera* sp. D18 genome.

| Biosynthetic cluster | Type | Start position | End position |
| --- | --- | --- | --- |
| Cluster 1 | hserlactone | 629,688 | 649,322 |
| Cluster 2 | hserlactone | 1,871,465 | 1,892,328 |
| Cluster 3 | arylpolyene | 3,122,221 | 3,183,631 |

**TABLE S3** Genes annotated in GEI-D18A.

| Start | End | Strand | Name | Gene | Function |
| --- | --- | --- | --- | --- | --- |
| 3200681 | 3200890 | - | E0Z06_RS15010 |  | resolvase |
| 3200822 | 3202537 | + | E0Z06_RS15015 |  | DDE-type integrase/transposase/recombinase |
| 3202540 | 3202992 | + | E0Z06_RS15020 | *tniB* | ATP-binding protein involved in transposition,TniB |
| 3203095 | 3203305 | + | E0Z06_RS15025 |  | winged helix-turn-helix transcriptional regulator |
| 3203312 | 3203382 | + | E0Z06_RS15030 |  | ArsR-family transcriptional regulator |
| 3203410 | 3204036 | - | E0Z06_RS15035 | *tetR* | TetR-family transcriptional regulator |
| 3204115 | 3205320 | + | E0Z06_RS15040 | *tet(B)* | tetracycline efflux MFS transporter Tet(B) |
| 3205433 | 3205942 | - | E0Z06_RS15045 |  | hypothetical protein |
| 3206044 | 3206475 | - | E0Z06_RS15050 |  | hypothetical protein |
| 3206494 | 3206673 | + | E0Z06_RS15055 |  | hypothetical protein |
| 3206603 | 3207442 | - | E0Z06_RS15060 | *sul1* | sulfonamide-resistant dihydropteroate synthase Sul1 |
| 3207436 | 3207783 | - | E0Z06_RS15065 | *qacE∆1* | quaternary ammonium compound efflux SMR transporter QacE delta 1 |
| 3207991 | 3208476 | - | E0Z06_RS15070 |  | hypothetical protein |
| 3208582 | 3209055 | - | E0Z06_RS15075 | *dfrA37* | trimethoprim-resistant dihydrofolate reductase DfrA |
| 3209124 | 3209678 | - | E0Z06_RS15080 | *aacA**3* | aminoglycoside N-acetyltransferase AAC(6')-IIa |
| 3209840 | 3210853 | + | E0Z06_RS15085 | *intI1* | class 1 integron integrase IntI1 |
| 3210819 | 3210998 | + | E0Z06_RS15090 |  | hypothetical protein |
| 3211131 | 3211337 | - | E0Z06_RS15095 |  | hypothetical protein |
| 3211422 | 3211830 | + | E0Z06_RS15100 |  | hypothetical protein |
| 3211833 | 3212825 | + | E0Z06_RS15105 | *tniB* | ATP-binding protein involved in transposition,TniB |
| 3212794 | 3213294 | - | E0Z06_RS15110 |  | GNAT-family N-acetyltransferase |
| 3213313 | 3213492 | + | E0Z06_RS15115 |  | hypothetical protein |
| 3213422 | 3214261 | - | E0Z06_RS15120 | *sul1* | sulfonamide-resistant dihydropteroate synthase Sul1 |
| 3214442 | 3214639 | + | E0Z06_RS15130 |  | cupin domain-containing protein |
| 3215208 | 3215849 | - | E0Z06_RS15135 | *catA* | type A chloramphenicol O-acetyltransferase |
| 3216600 | 3218141 | - | E0Z06_RS15145 | IS*91* | IS*91*-family transposase |
| 3218546 | 3219385 | - | E0Z06_RS15150 | *sul1* | sulfonamide-resistant dihydropteroate synthase Sul1 |
| 3219379 | 3219726 | - | E0Z06_RS15155 | *qacE∆1* | quaternary ammonium compound efflux SMR transporter QacE delta 1 |
| 3219890 | 3220681 | - | E0Z06_RS15160 | *aadA1* | ANT(3'')-Ia family aminoglycoside nucleotidyltransferase AadA1 |
| 3220827 | 3221840 | + | E0Z06_RS15165 | *intI1* | class 1 integron integrase IntI1 |
| 3221809 | 3222063 | + | E0Z06_RS15170 | Tn*3* | Tn*3*-family transposase |
| 3222018 | 3222752 | + | E0Z06_RS15175 |  | hypothetical protein |
| 3223266 | 3224288 | + | E0Z06_RS15180 | IS*21* | IS*21*-family transposase |
| 3224285 | 3225067 | + | E0Z06_RS15185 |  | AAA-family ATPase |
| 3225324 | 3225707 | - | E0Z06_RS15190 |  | hypothetical protein |
| 3225911 | 3226474 | + | E0Z06_RS15195 |  | recombinase-family protein |


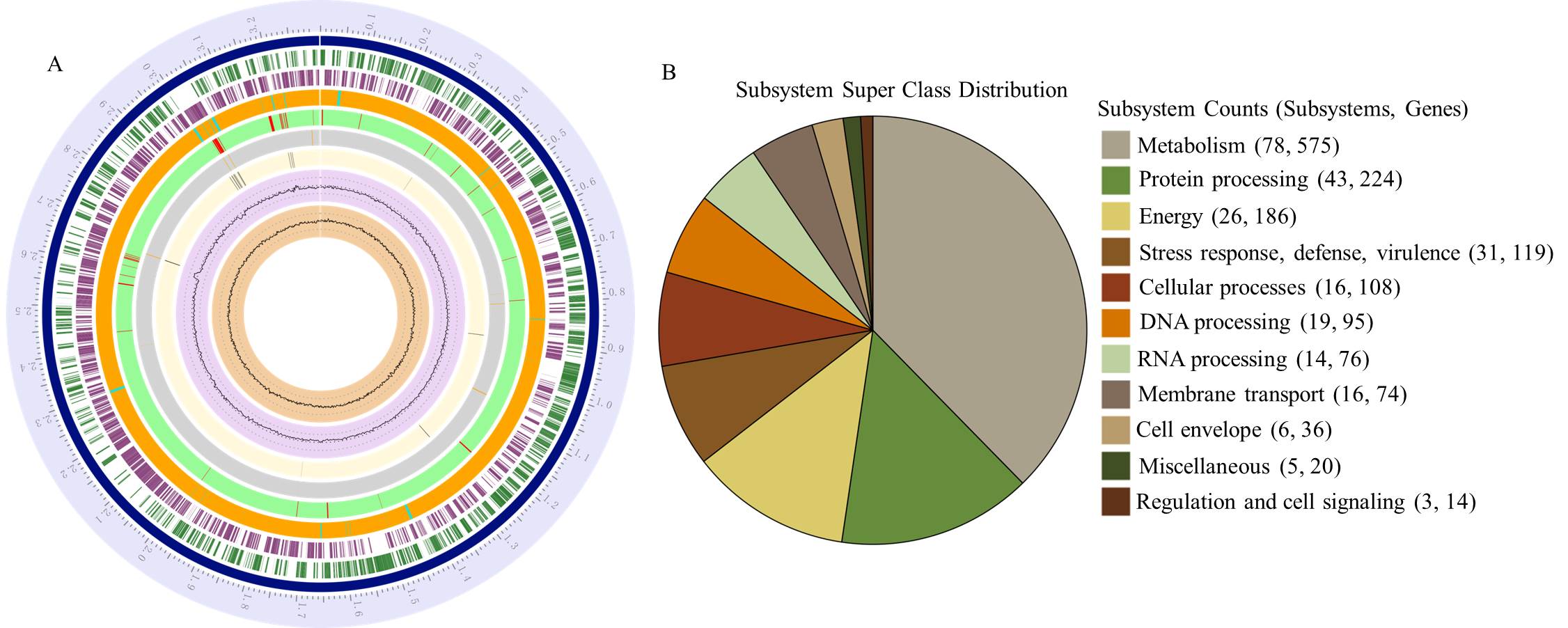


**FIGURE S1** Comprehensive genomic analysis of *Rheinheimera* sp. D18. **(A)** Circular graphical display of genomic features. From outer to inner rings: position label (Mbp); contigs/chromosome; coding sequences (CDS) on the forward strand; CDS on the reverse strand; non-CDS features; CDS with homology to known antimicrobial resistance genes; virulence factor genes; GC content and GC skew. **(B)** An overview of the subsystems associated with the D18 genome content. Gene classification according to subsystem is indicated by color.
